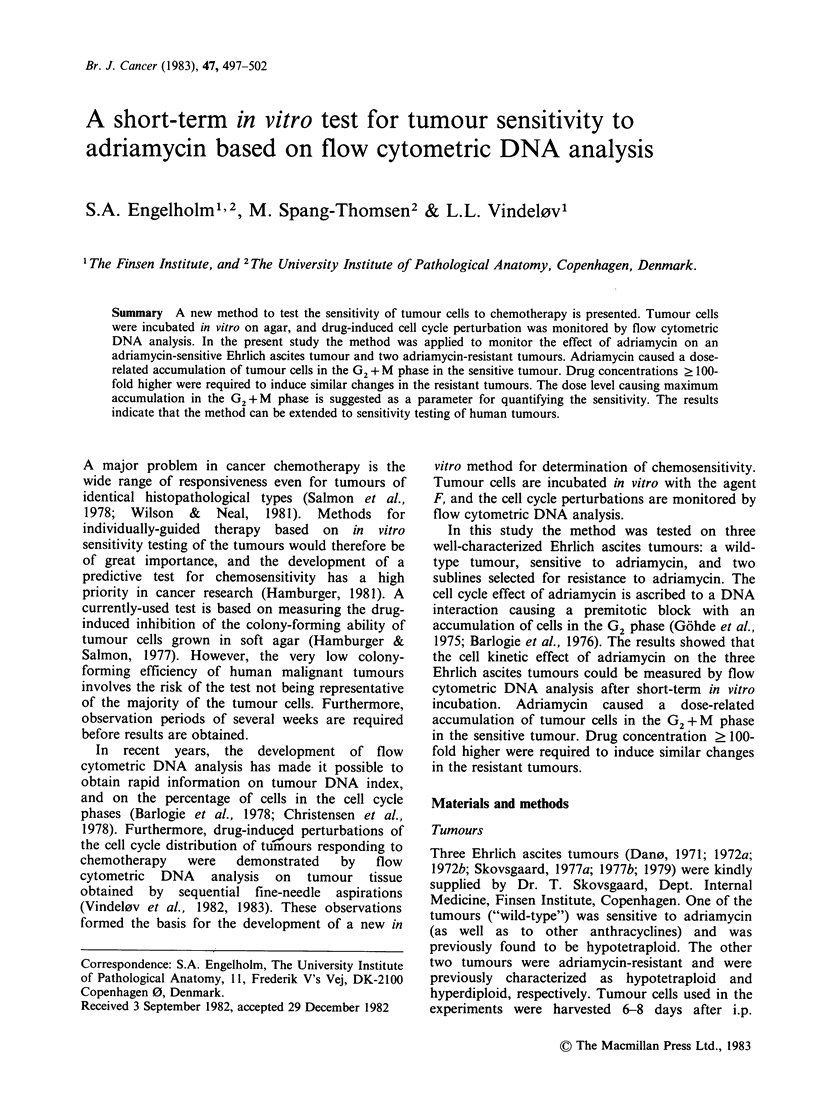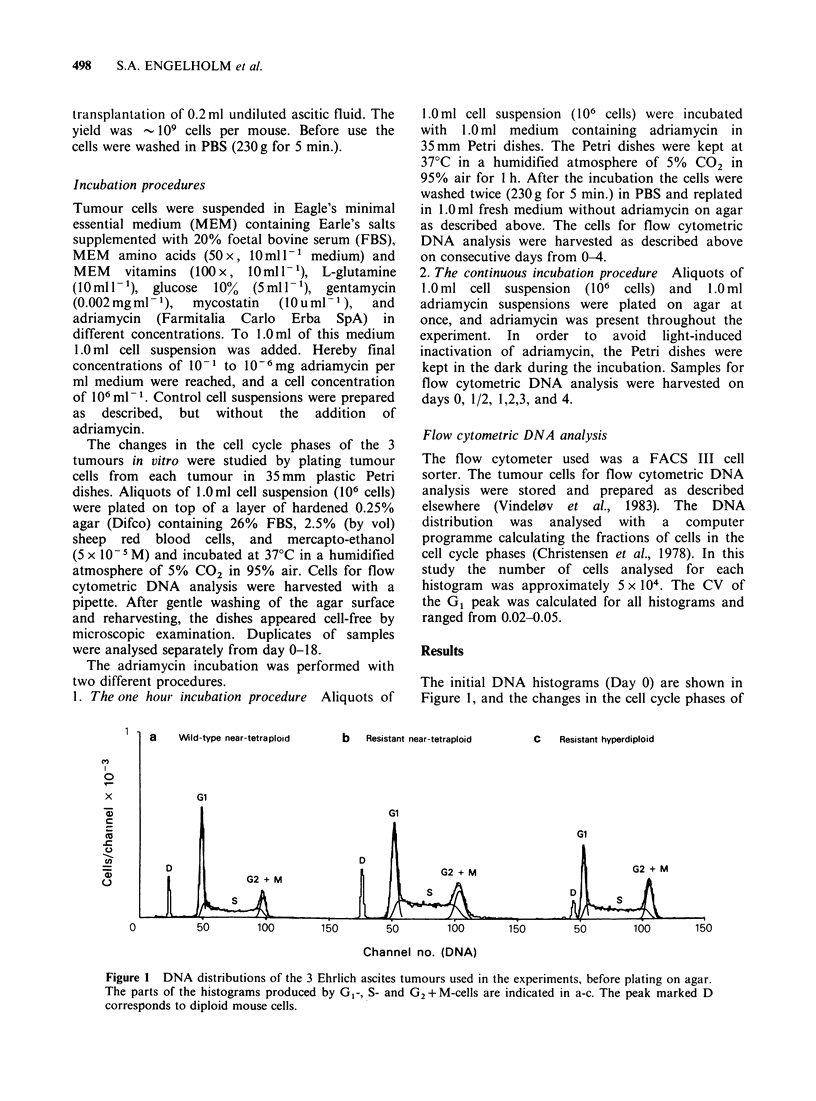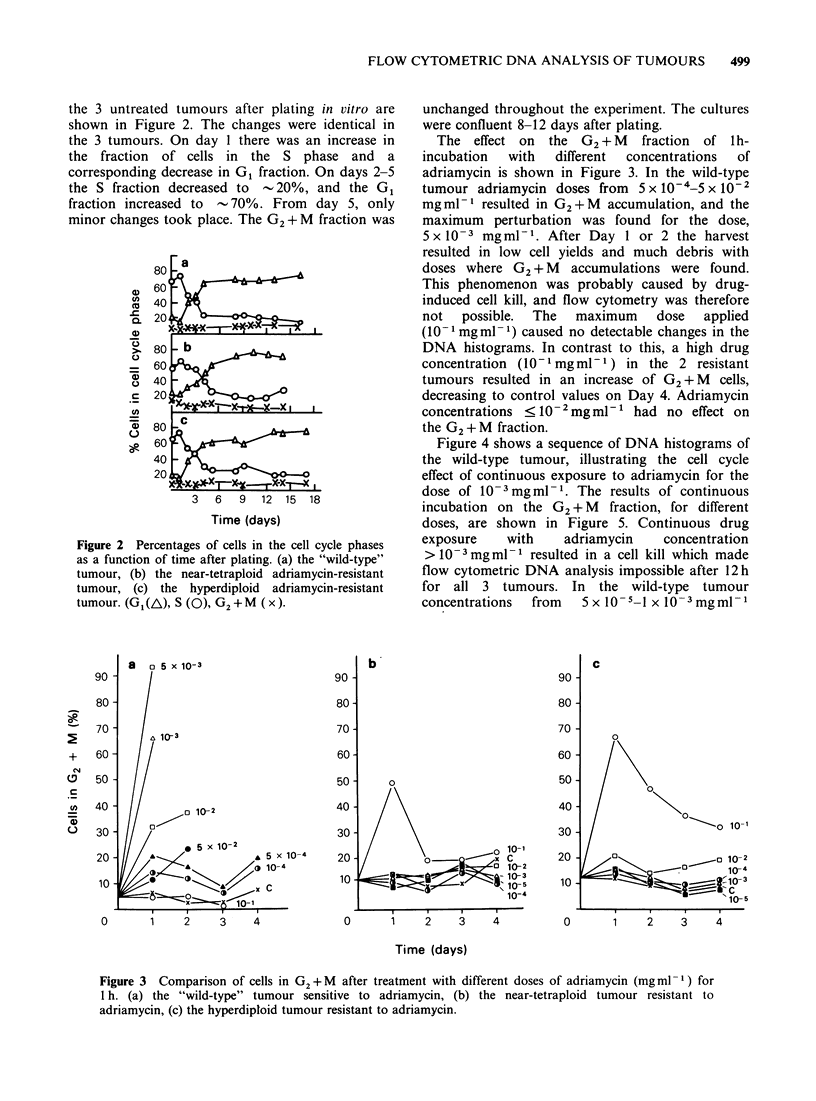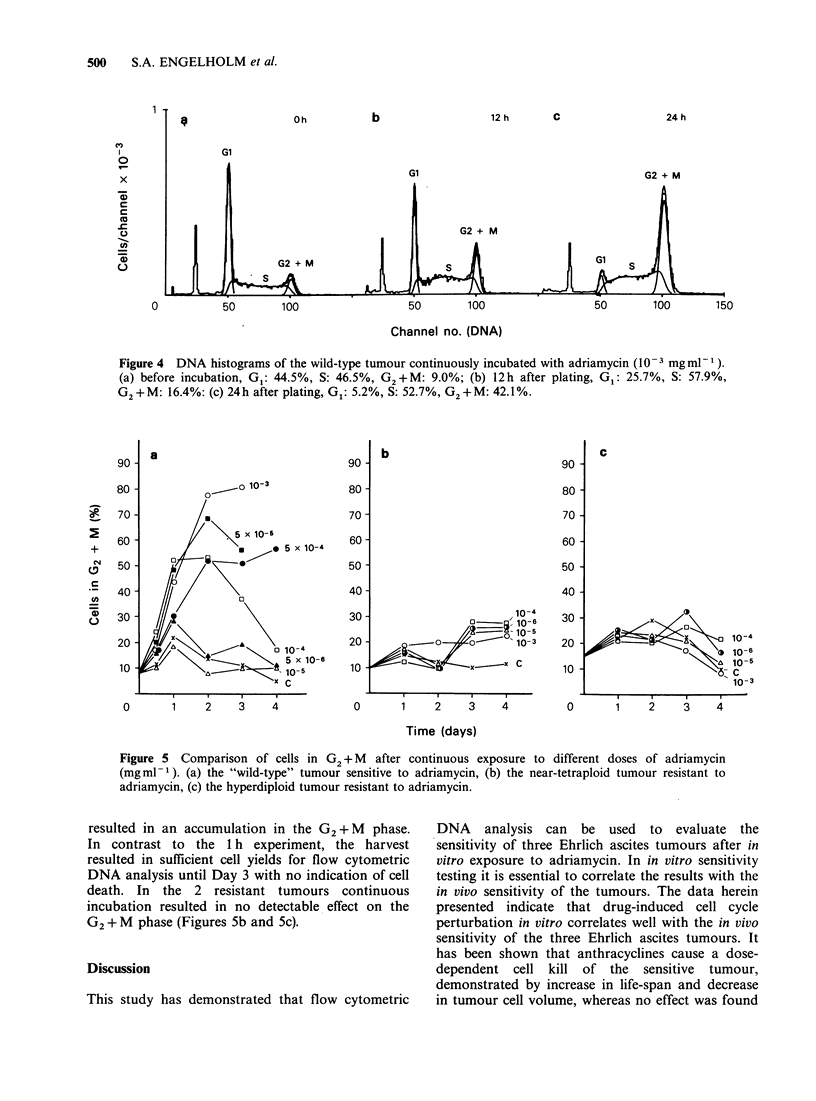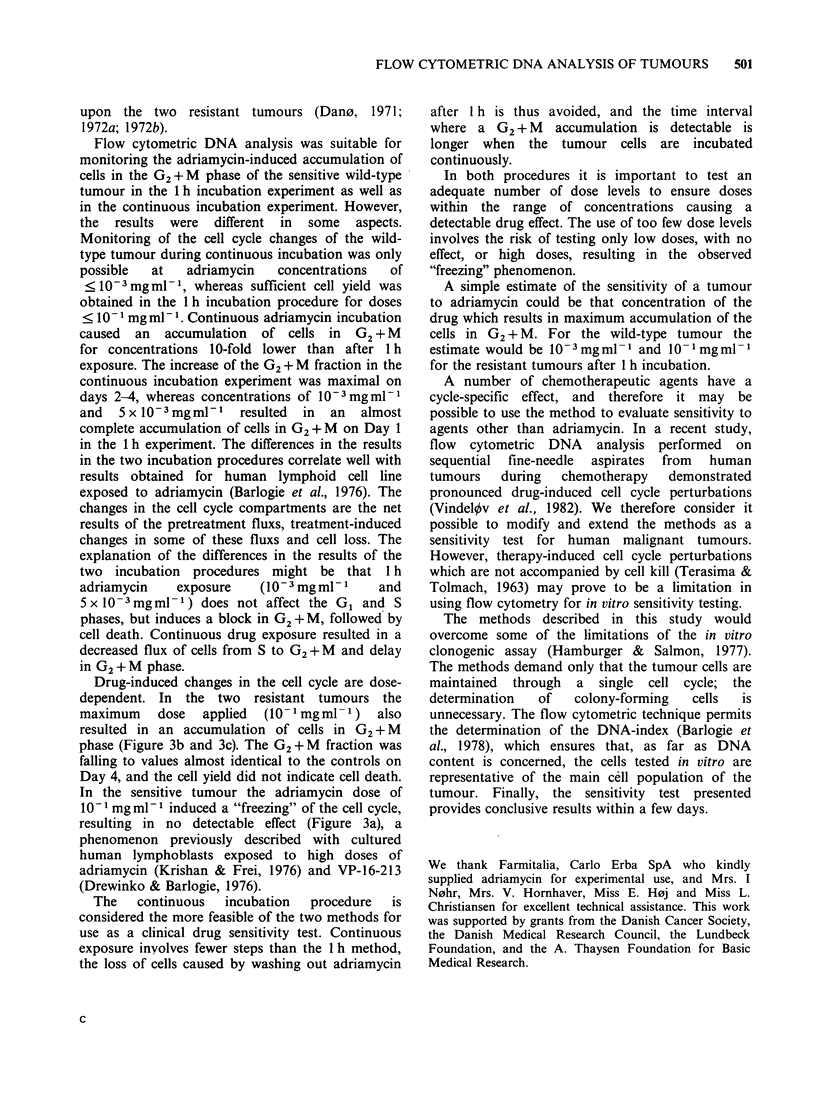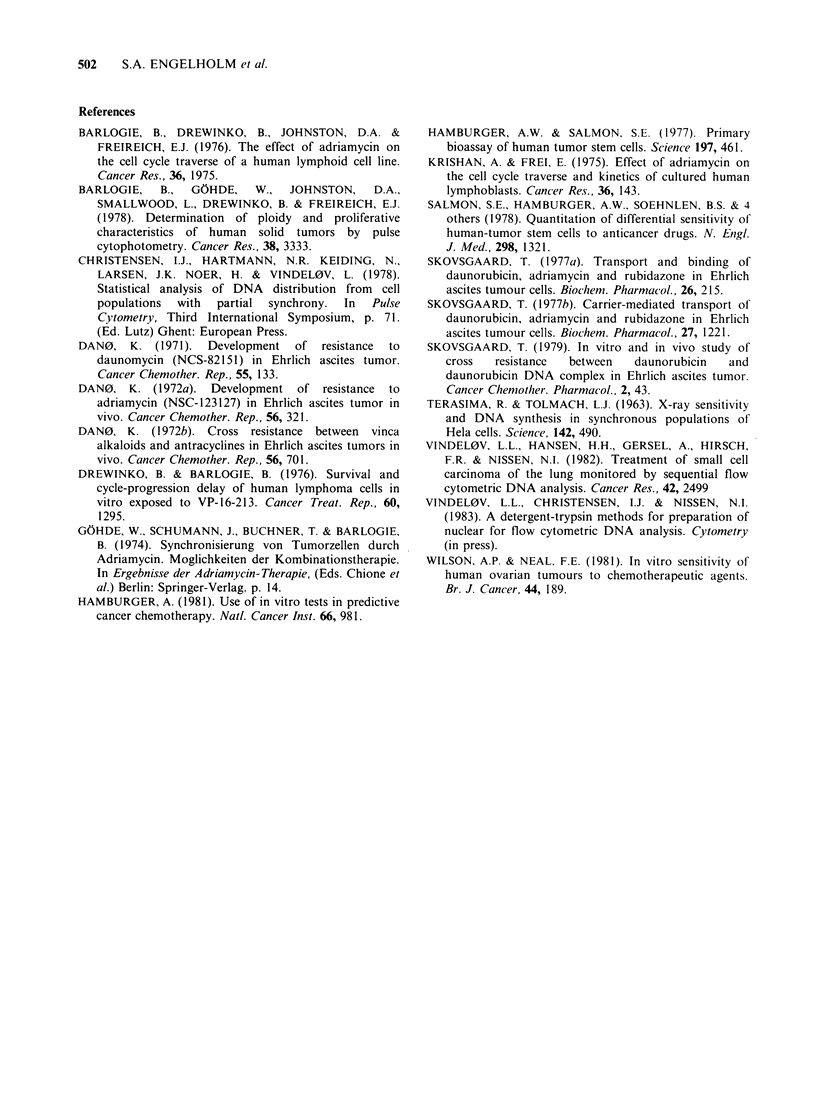# A short-term in vitro test for tumour sensitivity to adriamycin based on flow cytometric DNA analysis.

**DOI:** 10.1038/bjc.1983.79

**Published:** 1983-04

**Authors:** S. A. Engelholm, M. Spang-Thomsen, L. L. Vindeløv

## Abstract

A new method to test the sensitivity of tumour cells to chemotherapy is presented. Tumour cells were incubated in vitro on agar, and drug-induced cell cycle perturbation was monitored by flow cytometric DNA analysis. In the present study the method was applied to monitor the effect of adriamycin on an adriamycin-sensitive Ehrlich ascites tumour and two adriamycin-resistant tumours. Adriamycin caused a dose-related accumulation of tumour cells in the G2 + M phase in the sensitive tumour. Drug concentrations greater than or equal to 100-fold higher were required to induce similar changes in the resistant tumours. The dose level causing maximum accumulation in the G2 + M phase is suggested as a parameter for quantifying the sensitivity. The results indicate that the method can be extended to sensitivity testing of human tumours.


Br. J. Cancer (1983), 47, 497–502

### A short-term *in vitro* test for tumour sensitivity to adriamycin based on flow cytometric DNA analysis

S.A. Engelholm[1,2], M. Spang-Thomsen[2] & L.L. Vindeløv[1]

[1]*The Finsen Institute, and* [2]*The University Institute of Pathological Anatomy, Copenhagen, Denmark.*

**Summary** A new method to test the sensitivity of tumour cells to chemotherapy is presented. Tumour cells were incubated *in vitro* on agar, and drug-induced cell cycle perturbation was monitored by flow cytometric DNA analysis. In the present study the method was applied to monitor the effect of adriamycin on an adriamycin-sensitive Ehrlich ascites tumour and two adriamycin-resistant tumours. Adriamycin caused a dose-related accumulation of tumour cells in the $G_2+M$ phase in the sensitive tumour. Drug concentrations $\geq 100$-fold higher were required to induce similar changes in the resistant tumours. The dose level causing maximum accumulation in the $G_2+M$ phase is suggested as a parameter for quantifying the sensitivity. The results indicate that the method can be extended to sensitivity testing of human tumours.

A major problem in cancer chemotherapy is the wide range of responsiveness even for tumours of identical histopathological types (Salmon *et al.*, 1978; Wilson & Neal, 1981). Methods for individually-guided therapy based on *in vitro* sensitivity testing of the tumours would therefore be of great importance, and the development of a predictive test for chemosensitivity has a high priority in cancer research (Hamburger, 1981). A currently-used test is based on measuring the drug-induced inhibition of the colony-forming ability of tumour cells grown in soft agar (Hamburger & Salmon, 1977). However, the very low colony-forming efficiency of human malignant tumours involves the risk of the test not being representative of the majority of the tumour cells. Furthermore, observation periods of several weeks are required before results are obtained.

In recent years, the development of flow cytometric DNA analysis has made it possible to obtain rapid information on tumour DNA index, and on the percentage of cells in the cell cycle phases (Barlogie *et al.*, 1978; Christensen *et al.*, 1978). Furthermore, drug-induced perturbations of the cell cycle distribution of tumours responding to chemotherapy were demonstrated by flow cytometric DNA analysis on tumour tissue obtained by sequential fine-needle aspirations (Vindeløv *et al.*, 1982, 1983). These observations formed the basis for the development of a new *in vitro* method for determination of chemosensitivity. Tumour cells are incubated *in vitro* with the agent *F*, and the cell cycle perturbations are monitored by flow cytometric DNA analysis.

In this study the method was tested on three well-characterized Ehrlich ascites tumours: a wild-type tumour, sensitive to adriamycin, and two sublines selected for resistance to adriamycin. The cell cycle effect of adriamycin is ascribed to a DNA interaction causing a premitotic block with an accumulation of cells in the $G_2$ phase (Göhde *et al.*, 1975; Barlogie *et al.*, 1976). The results showed that the cell kinetic effect of adriamycin on the three Ehrlich ascites tumours could be measured by flow cytometric DNA analysis after short-term *in vitro* incubation. Adriamycin caused a dose-related accumulation of tumour cells in the $G_2+M$ phase in the sensitive tumour. Drug concentration $\geq 100$-fold higher were required to induce similar changes in the resistant tumours.

### Materials and methods

### *Tumours*

Three Ehrlich ascites tumours (Danø, 1971; 1972*a*; 1972*b*; Skovsgaard, 1977*a*; 1977*b*; 1979) were kindly supplied by Dr. T. Skovsgaard, Dept. Internal Medicine, Finsen Institute, Copenhagen. One of the tumours ("wild-type") was sensitive to adriamycin (as well as to other anthracyclines) and was previously found to be hypotetraploid. The other two tumours were adriamycin-resistant and were previously characterized as hypotetraploid and hyperdiploid, respectively. Tumour cells used in the experiments were harvested 6–8 days after i.p.

Correspondence: S.A. Engelholm, The University Institute of Pathological Anatomy, 11, Frederik V's Vej, DK-2100 Copenhagen Ø, Denmark.
Received 3 September 1982, accepted 29 December 1982

© The Macmillan Press Ltd., 1983

transplantation of 0.2 ml undiluted ascitic fluid. The yield was $\sim 10^9$ cells per mouse. Before use the cells were washed in PBS (230 g for 5 min.).

*Incubation procedures*

Tumour cells were suspended in Eagle's minimal essential medium (MEM) containing Earle's salts supplemented with 20% foetal bovine serum (FBS), MEM amino acids (50×, 10 ml $l^{-1}$ medium) and MEM vitamins (100×, 10 ml $l^{-1}$), L-glutamine (10 ml $l^{-1}$), glucose 10% (5 ml $l^{-1}$), gentamycin (0.002 mg $ml^{-1}$), mycostatin (10 u $ml^{-1}$), and adriamycin (Farmitalia Carlo Erba SpA) in different concentrations. To 1.0 ml of this medium 1.0 ml cell suspension was added. Hereby final concentrations of $10^{-1}$ to $10^{-6}$ mg adriamycin per ml medium were reached, and a cell concentration of $10^6$ $ml^{-1}$. Control cell suspensions were prepared as described, but without the addition of adriamycin.

The changes in the cell cycle phases of the 3 tumours *in vitro* were studied by plating tumour cells from each tumour in 35 mm plastic Petri dishes. Aliquots of 1.0 ml cell suspension ($10^6$ cells) were plated on top of a layer of hardened 0.25% agar (Difco) containing 26% FBS, 2.5% (by vol) sheep red blood cells, and mercapto-ethanol ($5 \times 10^{-5}$ M) and incubated at 37°C in a humidified atmosphere of 5% $CO_2$ in 95% air. Cells for flow cytometric DNA analysis were harvested with a pipette. After gentle washing of the agar surface and reharvesting, the dishes appeared cell-free by microscopic examination. Duplicates of samples were analysed separately from day 0–18.

The adriamycin incubation was performed with two different procedures.

1. *The one hour incubation procedure* Aliquots of 1.0 ml cell suspension ($10^6$ cells) were incubated with 1.0 ml medium containing adriamycin in 35 mm Petri dishes. The Petri dishes were kept at 37°C in a humidified atmosphere of 5% $CO_2$ in 95% air for 1 h. After the incubation the cells were washed twice (230 g for 5 min.) in PBS and replated in 1.0 ml fresh medium without adriamycin on agar as described above. The cells for flow cytometric DNA analysis were harvested as described above on consecutive days from 0–4.

2. *The continuous incubation procedure* Aliquots of 1.0 ml cell suspension ($10^6$ cells) and 1.0 ml adriamycin suspensions were plated on agar at once, and adriamycin was present throughout the experiment. In order to avoid light-induced inactivation of adriamycin, the Petri dishes were kept in the dark during the incubation. Samples for flow cytometric DNA analysis were harvested on days 0, 1/2, 1,2,3, and 4.

*Flow cytometric DNA analysis*

The flow cytometer used was a FACS III cell sorter. The tumour cells for flow cytometric DNA analysis were stored and prepared as described elsewhere (Vindeløv *et al.*, 1983). The DNA distribution was analysed with a computer programme calculating the fractions of cells in the cell cycle phases (Christensen *et al.*, 1978). In this study the number of cells analysed for each histogram was approximately $5 \times 10^4$. The CV of the $G_1$ peak was calculated for all histograms and ranged from 0.02–0.05.

### Results

The initial DNA histograms (Day 0) are shown in Figure 1, and the changes in the cell cycle phases of

**Figure 1** DNA distributions of the 3 Ehrlich ascites tumours used in the experiments, before plating on agar. The parts of the histograms produced by $G_1$-, S- and $G_2$+M-cells are indicated in a-c. The peak marked D corresponds to diploid mouse cells.

the 3 untreated tumours after plating *in vitro* are shown in Figure 2. The changes were identical in the 3 tumours. On day 1 there was an increase in the fraction of cells in the S phase and a corresponding decrease in $G_1$ fraction. On days 2–5 the S fraction decreased to $\sim$20%, and the $G_1$ fraction increased to $\sim$70%. From day 5, only minor changes took place. The $G_2+M$ fraction was

**Figure 2** Percentages of cells in the cell cycle phases as a function of time after plating. (a) the "wild-type" tumour, (b) the near-tetraploid adriamycin-resistant tumour, (c) the hyperdiploid adriamycin-resistant tumour. ($G_1$(△), S (○), $G_2+M$ (×).

unchanged throughout the experiment. The cultures were confluent 8–12 days after plating.

The effect on the $G_2+M$ fraction of 1h-incubation with different concentrations of adriamycin is shown in Figure 3. In the wild-type tumour adriamycin doses from $5\times10^{-4}$–$5\times10^{-2}$ mg ml$^{-1}$ resulted in $G_2+M$ accumulation, and the maximum perturbation was found for the dose, $5\times10^{-3}$ mg ml$^{-1}$. After Day 1 or 2 the harvest resulted in low cell yields and much debris with doses where $G_2+M$ accumulations were found. This phenomenon was probably caused by drug-induced cell kill, and flow cytometry was therefore not possible. The maximum dose applied ($10^{-1}$ mg ml$^{-1}$) caused no detectable changes in the DNA histograms. In contrast to this, a high drug concentration ($10^{-1}$ mg ml$^{-1}$) in the 2 resistant tumours resulted in an increase of $G_2+M$ cells, decreasing to control values on Day 4. Adriamycin concentrations $\leq10^{-2}$ mg ml$^{-1}$ had no effect on the $G_2+M$ fraction.

Figure 4 shows a sequence of DNA histograms of the wild-type tumour, illustrating the cell cycle effect of continuous exposure to adriamycin for the dose of $10^{-3}$ mg ml$^{-1}$. The results of continuous incubation on the $G_2+M$ fraction, for different doses, are shown in Figure 5. Continuous drug exposure with adriamycin concentration $>10^{-3}$ mg ml$^{-1}$ resulted in a cell kill which made flow cytometric DNA analysis impossible after 12 h for all 3 tumours. In the wild-type tumour concentrations from $5\times10^{-5}$–$1\times10^{-3}$ mg ml$^{-1}$

**Figure 3** Comparison of cells in $G_2+M$ after treatment with different doses of adriamycin (mg ml$^{-1}$) for 1 h. (a) the "wild-type" tumour sensitive to adriamycin, (b) the near-tetraploid tumour resistant to adriamycin, (c) the hyperdiploid tumour resistant to adriamycin.

**Figure 4** DNA histograms of the wild-type tumour continuously incubated with adriamycin ($10^{-3}$ mg ml$^{-1}$). (a) before incubation, $G_1$: 44.5%, S: 46.5%, $G_2$+M: 9.0%; (b) 12 h after plating, $G_1$: 25.7%, S: 57.9%, $G_2$+M: 16.4%: (c) 24 h after plating, $G_1$: 5.2%, S: 52.7%, $G_2$+M: 42.1%.

**Figure 5** Comparison of cells in $G_2$+M after continuous exposure to different doses of adriamycin (mg ml$^{-1}$). (a) the "wild-type" tumour sensitive to adriamycin, (b) the near-tetraploid tumour resistant to adriamycin, (c) the hyperdiploid tumour resistant to adriamycin.

resulted in an accumulation in the $G_2$+M phase. In contrast to the 1 h experiment, the harvest resulted in sufficient cell yields for flow cytometric DNA analysis until Day 3 with no indication of cell death. In the 2 resistant tumours continuous incubation resulted in no detectable effect on the $G_2$+M phase (Figures 5b and 5c).

### Discussion

This study has demonstrated that flow cytometric DNA analysis can be used to evaluate the sensitivity of three Ehrlich ascites tumours after *in vitro* exposure to adriamycin. In *in vitro* sensitivity testing it is essential to correlate the results with the *in vivo* sensitivity of the tumours. The data herein presented indicate that drug-induced cell cycle perturbation *in vitro* correlates well with the *in vivo* sensitivity of the three Ehrlich ascites tumours. It has been shown that anthracyclines cause a dose-dependent cell kill of the sensitive tumour, demonstrated by increase in life-span and decrease in tumour cell volume, whereas no effect was found upon the two resistant tumours (Danø, 1971; 1972*a*; 1972*b*).

Flow cytometric DNA analysis was suitable for monitoring the adriamycin-induced accumulation of cells in the $G_2+M$ phase of the sensitive wild-type tumour in the 1 h incubation experiment as well as in the continuous incubation experiment. However, the results were different in some aspects. Monitoring of the cell cycle changes of the wild-type tumour during continuous incubation was only possible at adriamycin concentrations of $\leq 10^{-3}$ mg ml$^{-1}$, whereas sufficient cell yield was obtained in the 1 h incubation procedure for doses $\leq 10^{-1}$ mg ml$^{-1}$. Continuous adriamycin incubation caused an accumulation of cells in $G_2+M$ for concentrations 10-fold lower than after 1 h exposure. The increase of the $G_2+M$ fraction in the continuous incubation experiment was maximal on days 2–4, whereas concentrations of $10^{-3}$ mg ml$^{-1}$ and $5\times10^{-3}$ mg ml$^{-1}$ resulted in an almost complete accumulation of cells in $G_2+M$ on Day 1 in the 1 h experiment. The differences in the results in the two incubation procedures correlate well with results obtained for human lymphoid cell line exposed to adriamycin (Barlogie *et al.*, 1976). The changes in the cell cycle compartments are the net results of the pretreatment fluxs, treatment-induced changes in some of these fluxs and cell loss. The explanation of the differences in the results of the two incubation procedures might be that 1 h adriamycin exposure ($10^{-3}$ mg ml$^{-1}$ and $5\times10^{-3}$ mg ml$^{-1}$) does not affect the $G_1$ and S phases, but induces a block in $G_2+M$, followed by cell death. Continuous drug exposure resulted in a decreased flux of cells from S to $G_2+M$ and delay in $G_2+M$ phase.

Drug-induced changes in the cell cycle are dose-dependent. In the two resistant tumours the maximum dose applied ($10^{-1}$ mg ml$^{-1}$) also resulted in an accumulation of cells in $G_2+M$ phase (Figure 3b and 3c). The $G_2+M$ fraction was falling to values almost identical to the controls on Day 4, and the cell yield did not indicate cell death. In the sensitive tumour the adriamycin dose of $10^{-1}$ mg ml$^{-1}$ induced a "freezing" of the cell cycle, resulting in no detectable effect (Figure 3a), a phenomenon previously described with cultured human lymphoblasts exposed to high doses of adriamycin (Krishan & Frei, 1976) and VP-16-213 (Drewinko & Barlogie, 1976).

The continuous incubation procedure is considered the more feasible of the two methods for use as a clinical drug sensitivity test. Continuous exposure involves fewer steps than the 1 h method, the loss of cells caused by washing out adriamycin after 1 h is thus avoided, and the time interval where a $G_2+M$ accumulation is detectable is longer when the tumour cells are incubated continuously.

In both procedures it is important to test an adequate number of dose levels to ensure doses within the range of concentrations causing a detectable drug effect. The use of too few dose levels involves the risk of testing only low doses, with no effect, or high doses, resulting in the observed "freezing" phenomenon.

A simple estimate of the sensitivity of a tumour to adriamycin could be that concentration of the drug which results in maximum accumulation of the cells in $G_2+M$. For the wild-type tumour the estimate would be $10^{-3}$ mg ml$^{-1}$ and $10^{-1}$ mg ml$^{-1}$ for the resistant tumours after 1 h incubation.

A number of chemotherapeutic agents have a cycle-specific effect, and therefore it may be possible to use the method to evaluate sensitivity to agents other than adriamycin. In a recent study, flow cytometric DNA analysis performed on sequential fine-needle aspirates from human tumours during chemotherapy demonstrated pronounced drug-induced cell cycle perturbations (Vindeløv *et al.*, 1982). We therefore consider it possible to modify and extend the methods as a sensitivity test for human malignant tumours. However, therapy-induced cell cycle perturbations which are not accompanied by cell kill (Terasima & Tolmach, 1963) may prove to be a limitation in using flow cytometry for *in vitro* sensitivity testing.

The methods described in this study would overcome some of the limitations of the *in vitro* clonogenic assay (Hamburger & Salmon, 1977). The methods demand only that the tumour cells are maintained through a single cell cycle; the determination of colony-forming cells is unnecessary. The flow cytometric technique permits the determination of the DNA-index (Barlogie *et al.*, 1978), which ensures that, as far as DNA content is concerned, the cells tested *in vitro* are representative of the main cell population of the tumour. Finally, the sensitivity test presented provides conclusive results within a few days.

We thank Farmitalia, Carlo Erba SpA who kindly supplied adriamycin for experimental use, and Mrs. I Nøhr, Mrs. V. Hornhaver, Miss E. Høj and Miss L. Christiansen for excellent technical assistance. This work was supported by grants from the Danish Cancer Society, the Danish Medical Research Council, the Lundbeck Foundation, and the A. Thaysen Foundation for Basic Medical Research.

### References

BARLOGIE, B., DREWINKO, B., JOHNSTON, D.A. & FREIREICH, E.J. (1976). The effect of adriamycin on the cell cycle traverse of a human lymphoid cell line. *Cancer Res.*, **36**, 1975.

BARLOGIE, B., GÖHDE, W., JOHNSTON, D.A., SMALLWOOD, L., DREWINKO, B. & FREIREICH, E.J. (1978). Determination of ploidy and proliferative characteristics of human solid tumors by pulse cytophotometry. *Cancer Res.*, **38**, 3333.

CHRISTENSEN, I.J., HARTMANN, N.R. KEIDING, N., LARSEN, J.K. NOER, H. & VINDELØV, L. (1978). Statistical analysis of DNA distribution from cell populations with partial synchrony. In *Pulse Cytometry,* Third International Symposium, p. 71. (Ed. Lutz) Ghent: European Press.

DANØ, K. (1971). Development of resistance to daunomycin (NCS-82151) in Ehrlich ascites tumor. *Cancer Chemother. Rep.*, **55**, 133.

DANØ, K. (1972*a*). Development of resistance to adriamycin (NSC-123127) in Ehrlich ascites tumor in vivo. *Cancer Chemother. Rep.*, **56**, 321.

DANØ, K. (1972*b*). Cross resistance between vinca alkaloids and antracyclines in Ehrlich ascites tumors in vivo. *Cancer Chemother. Rep.*, **56**, 701.

DREWINKO, B. & BARLOGIE, B. (1976). Survival and cycle-progression delay of human lymphoma cells in vitro exposed to VP-16-213. *Cancer Treat. Rep.*, **60**, 1295.

GÖHDE, W., SCHUMANN, J., BUCHNER, T. & BARLOGIE, B. (1974). Synchronisierung von Tumorzellen durch Adriamycin. Moglichkeiten der Kombinationstherapie. In *Ergebnisse der Adriamycin-Therapie,* (Eds. Chione *et al.*) Berlin: Springer-Verlag. p. 14.

HAMBURGER, A. (1981). Use of in vitro tests in predictive cancer chemotherapy. *Natl. Cancer Inst.* **66**, 981.

HAMBURGER, A.W. & SALMON, S.E. (1977). Primary bioassay of human tumor stem cells. *Science* **197**, 461.

KRISHAN, A. & FREI, E. (1975). Effect of adriamycin on the cell cycle traverse and kinetics of cultured human lymphoblasts. *Cancer Res.*, **36**, 143.

SALMON, S.E., HAMBURGER, A.W., SOEHNLEN, B.S. & 4 others (1978). Quantitation of differential sensitivity of human-tumor stem cells to anticancer drugs. *N. Engl. J. Med.*, **298**, 1321.

SKOVSGAARD, T. (1977*a*). Transport and binding of daunorubicin, adriamycin and rubidazone in Ehrlich ascites tumour cells. *Biochem. Pharmacol.*, **26**, 215.

SKOVSGAARD, T. (1977*b*). Carrier-mediated transport of daunorubicin, adriamycin and rubidazone in Ehrlich ascites tumour cells. *Biochem. Pharmacol.*, **27**, 1221.

SKOVSGAARD, T. (1979). In vitro and in vivo study of cross resistance between daunorubicin and daunorubicin DNA complex in Ehrlich ascites tumor. *Cancer Chemother. Pharmacol.*, **2**, 43.

TERASIMA, R. & TOLMACH, L.J. (1963). X-ray sensitivity and DNA synthesis in synchronous populations of Hela cells. *Science*, **142**, 490.

VINDELØV, L.L., HANSEN, H.H., GERSEL, A., HIRSCH, F.R. & NISSEN, N.I. (1982). Treatment of small cell carcinoma of the lung monitored by sequential flow cytometric DNA analysis. *Cancer Res.*, **42**, 2499

VINDELØV, L.L., CHRISTENSEN, I.J. & NISSEN, N.I. (1983). A detergent-trypsin methods for preparation of nuclear for flow cytometric DNA analysis. *Cytometry* (in press).

WILSON, A.P. & NEAL, F.E. (1981). In vitro sensitivity of human ovarian tumours to chemotherapeutic agents. *Br. J. Cancer*, **44**, 189.